# Key Considerations When Addressing Physical Inactivity and Sedentary Behaviour in People with Asthma

**DOI:** 10.3390/jcm12185998

**Published:** 2023-09-15

**Authors:** Paola D. Urroz Guerrero, Joice M. Oliveira, Hayley Lewthwaite, Peter G. Gibson, Vanessa M. McDonald

**Affiliations:** 1 National Health and Medical Research Council, Centre of Excellence in Treatable Traits, Newcastle, NSW 2305, Australia; paola.urroz@newcastle.edu.au (P.D.U.G.); hayley.lewthwaite@newcastle.edu.au (H.L.); peter.gibson@newcastle.edu.au (P.G.G.); 2 Asthma and Breathing Program, Hunter Medical Research Institute, Newcastle, NSW 2305, Australia; joice.mara.oli@gmail.com; 3 School of Nursing and Midwifery, University of Newcastle, Newcastle, NSW 2308, Australia; 4 Graduate Program in Rehabilitation Sciences, Pitagoras Unopar University, Londrina 86041-140, PR, Brazil; 5 Laboratory of Research in Respiratory Physiotherapy, Department of Physiotherapy, State University of Londrina, Londrina 86038-350, PR, Brazil; 6 School of Medicine and Public Health, University of Newcastle, Newcastle, NSW 2308, Australia; 7 Department of Respiratory and Sleep Medicine, John Hunter Hospital, Newcastle, NSW 2305, Australia

**Keywords:** asthma, physical activity, sedentary behaviour

## Abstract

People with asthma tend to be less physically active and more sedentary than people without asthma. This narrative review aimed to present key considerations when addressing physical inactivity and sedentary behaviour in people with asthma by identifying barriers and facilitators, determinants and correlates, and intervention approaches. Using a search strategy, electronic databases were searched for relevant studies. Data extracted from studies were qualitatively synthesised. A total of 26 studies were included in the review. Six studies reported asthma symptoms as a barrier to physical activity, while four studies reported having a supportive network as a physical activity facilitator. Across studies, physical activity correlates/determinants were pulmonary function, exercise capacity, body mass index, dyspnoea, psychological health, and asthma control. Interventions that effectively improved physical activity in the short term were a step-based prescription programme, a weight loss programme incorporating aerobic and resistance training, and a weight loss lifestyle intervention, while a high-intensity interval training pulmonary rehabilitation program was effective in the long term. The collective findings suggest that a personalised physical activity programme incorporating different strategies is needed. There was minimal evidence to provide recommendations to optimise sedentary behaviour in asthma, and more research is needed on the topic.

## 1. Introduction

Asthma is a common chronic respiratory condition estimated to affect 262 million people worldwide [1]. The disease burden from asthma is excessive, with about 1000 preventable asthma deaths per day globally [1]. Pharmacological stepwise therapy is the first line of treatment for asthma. This has advanced significantly with add-on therapies [2,3,4]. However, even on optimal pharmacotherapy treatment, people with asthma still live with a high disease burden, which negatively impacts participation in daily life activities and quality of life [5,6]. To address these unmet health needs, new asthma management strategies are needed [7,8]. “Treatable Traits” has been proposed as a personalised model of care for people with obstructive airway disease [9,10]. Treatable Traits involves identifying and treating clinically relevant disease characteristics (i.e., traits) for each individual to improve outcomes. Physical inactivity and sedentary behaviour have been identified as treatable traits in people living with asthma [11]. These behaviours are identifiable, clinically relevant, and treatable, meeting the essential criteria of a treatable trait [10].

**Figure 1:**
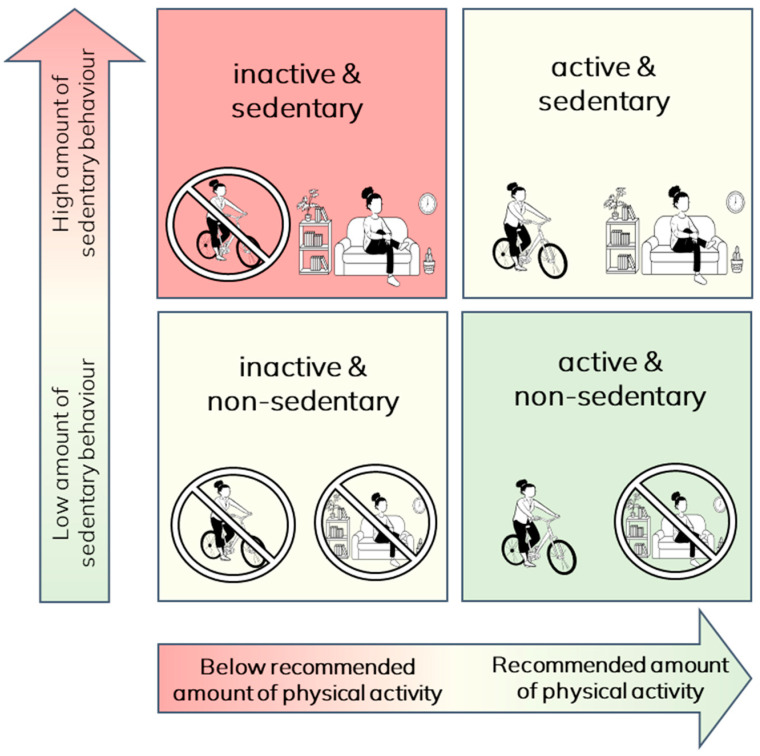
Movement behaviour profiles.

Daily life activities are typically categorised according to the amount of energy required to carry out the activity, often expressed as metabolic equivalents of task (METs), where 1 MET is 3.5 mL of oxygen per kg of body mass per minute and is the energy equivalent of sitting quietly. Physical activity is defined as ‘any bodily movement that is produced by the skeletal muscles that requires energy expenditure’ [12]. Physical activities can be light (>1.5 METs but <3 METs), moderate (≥3 METs but <6 METs), or vigorous intensity (≥6 METs). Sedentary behaviours are those that have a low energy requirement (≤1.5 METs) and are performed in the sitting, reclining, or lying positions during waking hours [13]. It is important to recognise that physical inactivity and sedentary behaviour are two separate constructs (it is possible to be inactive yet not sedentary, or active and sedentary) that require different intervention approaches [14] (Figure 1). Physical inactivity refers to not meeting the recommended amount of moderate to vigorous physical activity (MVPA). Sedentary refers to high proportions of wake time spent in sedentary behaviour. 

Compared to people without asthma, those with asthma are less likely to meet physical activity guideline recommendations (i.e., be inactive) [15,16,17,18,19], which has major health consequences. In the general population, there is a large body of evidence supporting the health benefits of meeting physical activity guidelines, including preventing cardiovascular disease, diabetes, cancer, hypertension, obesity, depression and osteoporosis [12,20]. Regular physical activity has also been shown to be associated with asthma-specific benefits, including improved exercise capacity, asthma control, pulmonary function, health-related quality of life (HRQoL), and lower levels of systemic inflammation [17,21]. 

People with asthma have also been shown to be sedentary, spending up to nine hours per day in sedentary behaviour [22]. Since the early 2000s, there has been increasing evidence to support the deleterious health effects of prolonged time spent in sedentary behaviour, including increased risk of diabetes, cardiovascular disease, and all-cause mortality [23,24]. Importantly, these health risks are independent of the time spent being physically active [12,24]. In people with asthma, while evidence in this space is still emerging, increased time spent being sedentary has been shown to be associated with reduced pulmonary function, asthma control and exercise capacity, and more healthcare utilisation including overnight hospital stays [17,25]. 

Given the impact these behaviours have on asthma and general health outcomes, addressing physical inactivity and sedentary behaviour is of critical importance; however, there are few recommendations for how to address these behaviours in current asthma treatment guidelines [26]. For example, although the 2023 Global Initiative on Asthma strategy document identifies physical activity as a non-pharmacological strategy to assist in improving asthma symptom control or reducing future risk [27], no specific recommendations are provided on how to improve physical activity and there is no mention of sedentary behaviour. This creates a barrier for healthcare providers for how to achieve meaningful changes to physical activity participation and sedentary behaviour as part of asthma management [14,28,29,30]. 

The aim of this narrative review was to present key considerations when addressing physical inactivity and sedentary behaviour in people with asthma. Specifically, this narrative review aimed to answer the following research questions about people with asthma:

(1) What are self-reported behavioural barriers and facilitators to increasing physical activity and reducing sedentary behaviour? (Aim 1)
(2) What are determinants and correlates of physical activity and sedentary behaviour? (Aim 2)
(3) What are effective intervention approaches for optimising physical activity and sedentary behaviour? (Aim 3)

## 2. Materials and Methods

This review was reported according to the Realist and Meta-narrative Evidence Synthesis: Evolving Standards project (RAMESES) [31]. The quality of reporting in this study follows the Scale for Assessment of Narrative Review Articles (SANRA) guidelines (Figure S1).

### 2.1. Data Sources and Search Strategy

A search strategy was developed by authors P.U.G. and J.M.O., with the assistance of an experienced academic librarian, for searching the electronic databases Ovid (EMBASE, Medline and Psycinfo), CINAHL (EBESCO), and Cochrane. Keywords related to asthma (asthma.mp or asthmatic.mp. or “bronchial asthma”.mp.), physical activity and sedentary behaviour (“sedentary behavio?r”.mp. or sedentarism.mp. or physical activit*.mp. or physical inactivit*.mp. or sitting.mp. or reclining.mp. or lying.mp. or seated.mp. or “screen time”.mp. or “daily life physical activit*”.mp. or “activit* daily living”.mp. or running.mp. or swimming.mp. or walking.mp. or stair climbing.mp) were included as a base search strategy. Additional keywords and terms were used to address the three different aims of the review: barriers and facilitators for Aim 1, correlates and determinants for Aim 2, and systematic reviews and interventions for Aim 3 (Table S1). Electronic databases were searched from inception to the 13 April 2023. 

### 2.2. Inclusion and Exclusion Criteria

Studies were included in this review if they met the following a priori defined criteria:

(1) Population: Adults ≥ 18 yrs with diagnosed asthma
(2) Outcomes of interest: For Aim 1, studies that aimed to explore barriers and facilitators to increasing physical activity and reducing sedentary behaviour. For Aims 2 and 3, studies that aimed to examine physical activity participation (steps per day, time spent in MVPA, etc.) and/or sedentary behaviour (sedentary time, sit-to-stand transitions, etc).
(3) Designs: For Aim 1, qualitative studies were included (focus groups, interviews, etc.). Quantitative studies were also included if a qualitative element was addressed (surveys, questionnaires etc). For Aim 2, cohort or cross-sectional studies were included. Studies that reported on correlation analyses were included to answer questions about physical activity and sedentary behaviour ‘correlates’. Studies that reported results from prediction analyses were included to answer questions about physical activity and sedentary behaviour ‘determinants’ where physical activity or sedentary behaviour outcomes were included in the model as the dependent variable. For Aim 3, systematic reviews with or without meta-analyses were included.
(4) Evaluations: For Aim 1, studies evaluating barriers and/or facilitators/enablers were included. For Aim 2, studies evaluating associations between physiological, psychological, socioeconomic, and/or behavioural factors and physical activity and/or sedentary behaviour were included. For Aim 3, systematic reviews including primary studies that evaluated the effects of pharmacological or non-pharmacological intervention on physical activity and/or sedentary behaviour outcomes were included.

Studies were excluded if they did not involve people with asthma or were not published in English. Notes, case studies, animal studies, narrative reviews, editorials, and scientific congress abstracts were also excluded. 

### 2.3. Study Extraction, Analysis, and Synthesis 

Titles and abstracts identified from the search process were independently screened for eligibility by two reviewers (P.U.G. and J.M.O.) in Covidence after duplicates were removed. For references where eligibility could not be determined, full-text articles were extracted and independently screened by two reviewers (P.U.G., J.M.O.). Data were extracted from all eligible studies and qualitatively synthesised for each aim. For Aim 3, the overlap of primary studies included within the eligible systematic reviews was calculated. A decision tool for an overview of reviews by Pollock et al. [32] was used to inform which systematic reviews were included in the qualitative synthesis. 

## 3. Results

### 3.1. Search Results

**Figure 2:**
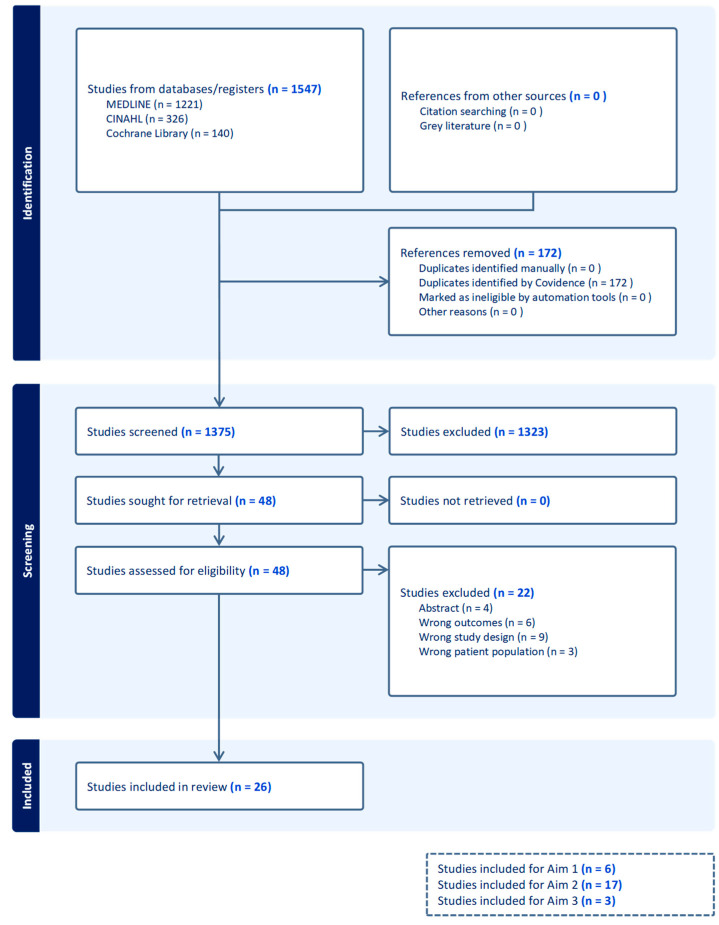
PRISMA flowchart.

A total of 1375 unique studies were identified in the search process. After title and abstract screening, 48 studies were screened for eligibility. A total of 26 studies were included in the review: six studies addressed Aim 1, 17 addressed Aim 2, and three addressed Aim 3. The results of the study selection process are summarised in a PRISMA flowchart (Figure 2). 

### 3.2. Characteristics of Studies

The characteristics of all included studies are summarised in Table 1.

#### 3.2.1. Aim 1: Barriers and Facilitators of Physical Activity and Sedentary Behaviour 

Six studies including 15–92 participants were included [33,34,35,36,37,38] that assessed barriers (k = 6) or facilitators (k = 4) to exercise and/or physical activity participation [33,35,36,38]. No study explored perceived influences of sedentary behaviour. Participants’ perspectives about physical activity were explored via one-on-one interviews [35,36,38], focus groups [37], a questionnaire [34], or topics of discussion in an asthma online community forum [33]. In two studies [35,38], participants’ perceptions were explored following a physical activity intervention. 

**Table 1:** Characteristics of included studies.

Reference	Country	Number of Participants/Studies	Asthma Severity	Study Design	Physical Activity Outcomes	Sedentary Behaviour Outcomes
Aim 1: Barriers and facilitators of physical activity and/or sedentary behaviour						
Mancuso et al. (2006) [36]	United States of America	60 participants (88% female)	Any severity	Cross-sectional qualitative study (interviews)	Open-ended questions about physical activity	No
Nyenhuis et al. (2019) [37]	United States of America	20 participants (100% female)	Any severity	Qualitative study (focus groups and interviews)	Questions to assess physical activity barriers	No
Freeman et al. (2020) [34]	United Kingdom	62 participants (66% female)	Difficult asthma	Cross-sectional cohort (Questionnaires)	Exercise therapy burden questionnaire	No
Hiles et al. (2021) [35]	Australia	13 participants (62% female)	Severe asthma	Qualitative study (Interviews)	Open-ended questions about physical activity	No
Attalla et al. (2022) [33]	United Kingdom	92 participants (36% female, 52% not stated)	Any severity	Qualitative analysis (Posts in online forum)	Posts about exercising with asthma	No
Papp et al. (2022) [38]	Sweden	8 participants (100% female)	Any severity	Qualitative study (Interviews)	Open-ended questions about physical activity	No
Aim 2: Correlates and determinants of physical activity and/or sedentary behaviour						
Mälkiä and Impivaara (1998) [39]	Finland	178 participants (58% female)	Any severity	Cross-sectional study	Questionnaire	No
Mancuso et al. (2007) [40]	United States of America	258 participants (75% female)	Mild to moderate	Cross-sectional study	Questionnaire	No
Dogra et al. (2008) [41]	Canada	4895 participants (66% female)	Any severity	Cross-sectional study	Questionnaire	No
Ramos et al. (2015) [42]	Brazil	20 participants (70% female)	Severe asthma	Prospective, cross-sectional, case–control study	Questionnaire	No
van’t Hul et al. (2016) [19]	Netherlands	226 participants (62% female)	Any severity	Prospective, cross-sectional study	Accelerometer	No
Good et al. (2017) [43]	Canada	2740 participants (62% female)	Any severity	Cross-sectional study	Questionnaire	No
Vermeulen et al. (2017) [44]	Belgium	20 participants (54% female)	Any severity	Prospective observational study	Accelerometer	No
Yamasaki et al. (2017) [45]	Japan	18 participants (56% female)	Any severity	Cross-sectional study	Accelerometer	No
Coelho et al. (2018) [46]	Brazil	36 participants (100% female)	Moderate to severe	Cross-sectional study	Pedometer	No
Hennegrave et al. (2018) [47]	France	51 participants (59% female)	Any severity	Prospective study	Accelerometer	No
Cordova-Rivera et al. (2019) [48]	Australia	62 participants (52% female)	Severe asthma	Cross-sectional study	Accelerometer	No
Abdo et al. (2021) [49]	Germany	268 participants (56% female)	Any severity	Cross-sectional study	Accelerometer	No
Cordova-Rivera et al. (2021) [22]	Australia	27 participants (56% female)	Severe asthma	Cross-sectional study	Accelerometer	Accelerometer *
Almatruk and Axon (2022) [50]	United States of America	2410 participants (58% female)	Any severity	Retrospective, cross-sectional study	Questionnaire	No
Hansen et al. (2022) [51]	Denmark	60 participants (53% female)	Any severity	Prospective follow-up study	Accelerometer	Yes
Ozsoy et al. (2022) [52]	Turkey	57 participants (91% female)	Any severity	Cross-sectional study	Questionnaire	No
Ricketts et al. (2023) [53]	United Kingdom	75 participants (56% female)	Mild to moderate asthma	Cross-sectional study	Accelerometer	Yes
Aim 3: Interventions for optimising physical activity and sedentary behaviour						
McLoughlin et al. (2022) [54]	Australia	4 studies	Moderate to severe asthma	Systematic review and meta-analysis	Accelerometers and pedometer	No
Osadnik et al. (2022) [55]	Australia	10 studies	Any severity	Systematic review and meta-analysis	Accelerometer	No
Tyson et al. (2022) [56]	United Kingdom	25 studies	Any severity	Systematic review	Accelerometers, pedometers and questionnaires	Accelerometers and questionnaires

* Measured sedentary behaviour as an outcome, but did not assess the correlates or determinants of sedentary behaviour.

#### 3.2.2. Aim 2: Correlates and Determinants of Physical Activity and Sedentary Behaviour 

A total of 17 studies explored correlates (k = 13) [19,39,40,42,43,44,45,46,47,49,51,52,53] or determinants (k = 6) [22,41,43,46,47,48,50] of physical activity or sedentary behaviour; most (82%) were of cross-sectional design (Table 2). Of the 17 studies, 10 (59%) assessed physical activity or sedentary behaviour with pedometers or accelerometer devices (Table 2). Most studies (k = 8, 47%) sought to determine whether physical activity was associated with measures of pulmonary function, followed by exercise capacity, asthma control, obesity or body mass index (BMI), eosinophilic inflammation, psychological health, dyspnoea, exacerbations, sociodemographic factors, quality of life (QoL), or self-perceived health. Only two studies analysed the relationship between physical activity and self-reported functional limitation, and one study analysed the relationship with medication use or fruit and vegetable intake. 

Two studies [51,53] reported correlations between health-related variables and sedentary behaviour, and no study sought to identify sedentary behaviour determinants. Both of the abovementioned studies reported correlations between sedentary behaviour and asthma control, pulmonary function, eosinophilic inflammation, airway inflammation or QoL [51,53]. Ricketts et al. [53] reported correlations between sedentary behaviour and exercise capacity, dyspnoea, body mass, psychological health, and medication usage.

#### 3.2.3. Aim 3: Interventions for Optimising Physical Activity and Sedentary Behaviour

Three systematic reviews were included to address Aim 3 [54,55,56], of which two included meta-analyses and one was a Cochrane review. Tyson et al. [56] included 10 studies that reported on intervention effects on physical activity, while three studies also included sedentary behaviour as an outcome. Osadnik et al. [55] and McLoughlin et al. [54] included one and four studies, respectively, that reported on intervention effects on physical activity. In the review by Tyson et al. [56], two studies were duplicated data [57,58,59,60] and there was overlap across reviews for studies included [57,58,61,62,63], resulting in nine unique studies across the three systematic reviews. Of these, seven were randomised controlled trials (RCT) [57,58,59,60,61,62,63,64,65], one was a feasibility study [66] and one was a non-RCT [67]. In five of the nine unique studies, the intervention was supervised exercise [57,58,61,62,63,64,65,66,67], two studies were of walking interventions [61,66] and two studies were behaviour change interventions [59,60,64]. 

### 3.3. Main Findings

#### 3.3.1. Aim 1: Barriers and Facilitators of Physical Activity and Sedentary Behaviour 

A broad list of physical activity barriers was identified by people with asthma. Asthma-related and general health-related barriers were articulated by participants in all six studies. Among this topic, participants reported fear of worsening of symptoms during exercise and/or being embarrassed by symptoms, fear of having an exacerbation, and believing that exercising can be dangerous. Lack of knowledge about exercising, self-monitoring, and benefits of physical activity was also a main theme [33,37]. Other barriers mentioned by participants were physical symptoms during and after exercise, such as pain, discomfort, or fatigue [33,34]. Psychological problems [33,36], motivational problems [34], and lack of support [37] were also highlighted by people with asthma as were external and environmental factors [33,36,37,38].

Regarding facilitators of physical activity, having a supportive network with healthcare professionals [33,35,36] and families and friends [35,36,38] was a common theme (k = 4). Other facilitators were positive thinking [35,36] or experiencing the positive effects of exercise [33], integrating physical activity or exercise into their routine, goal setting, self-care [36,38], the programme and structure of exercise sessions, and use of activity trackers [35].

#### 3.3.2. Aim 2: Correlates and Determinants of Physical Activity and Sedentary Behaviour

The clinical correlates and determinants of physical activity were found to be pulmonary function (r = 0.34 to 0.56; r^2^ = 0.06), exercise capacity (r = 0.38 to 0.61; r^2^ = 0.26), BMI (r^2^ = −0.12 to −0.29), dyspnoea (r = −0.39 to −0.30; r^2^ = 0.09 to 0.17), psychological health (r = −0.32 to 0.28), and asthma control (r = −0.46 to −0.20; r^2^ = 0.21) (Table 2). In addition, self-perceived health, self-reported functional limitation, and sociodemographic variables (e.g., age, gender, race, income, education) were also important determinants of physical activity in multivariate analyses that explained up to 51% of physical activity volume [43,47,50] (Table 2). 

Only one of four studies found a statistically significant correlation between physical activity and QoL [53]. Studies that assessed the correlation between physical activity and eosinophils [22,48,51,53], Immunoglobulin E (IgE) [51], and markers of oxidative stress [45] did not find statistically significant results. However, associations with physical activity were found for high-sensitivity C-reactive protein (r^2^ = 0.15) [22,48] and fractional concentration of exhaled nitric oxide (FeNo) (r = −0.37, *p* = 0.005) [53]. Exacerbations [43], medication use [53] and food intake [43] were investigated in only one study each; of those, only medication use was not correlated with physical activity.

Regarding sedentary behaviour, FeNo was associated with sedentary behaviour in two studies (r = 0.28 and 0.33). Rickets et al. [53] reported significant correlations between sedentary behaviour and exercise capacity (r = −0.57; *p* < 0.001), dyspnoea (r = 0.42; *p* = 0.001), BMI (r = 0.51; *p* < 0.001), inhaled corticosteroid dose (r = 0.48; *p* < 0.001), and number of prednisolone courses per year (r = 0.35; *p* = 0.009). Asthma control (r = 0.43, *p* = 0.001), pulmonary function (r = −0.50, *p* < 0.001), and QoL (r = −0.46, *p* < 0.001) were found to be significantly correlated with sedentary behaviour in Rickets et al. [53] but not by Hansen et al. [51]. Steps per day were negatively correlated with sedentary behaviour (r = −0.61, *p* < 0.05) by Hansen et al. [51]. No associations were apparent between sedentary behaviour and eosinophils [51,53], IgE [51], or anxiety and depression symptoms [53].

#### 3.3.3. Aim 3: Interventions for Optimising Physical Activity and Sedentary Behaviour

Four of the seven RCTs reported a significant improvement in physical activity following intervention compared with a control group. These interventions were: a step-based prescription programme using a pedometer (increase to 1558 versus decrease to 750 steps per day at 12 weeks (*p* = 0.005), no longer significant at 24–28 weeks) [61]; a weight loss programme with aerobic and resistance muscle training (increase to 18.2 min versus 7.9 min of MVPA per day (*p* < 0.001), increase to 3274 versus 729 steps per day (*p* < 0.001) and increase to 54.8 min versus decrease to 2.8 min of light intensity physical activity per day at 12 weeks (*p* = 0.03)) [58]; a weight loss lifestyle intervention (increase to 418.2 versus 178.8 MET minutes per week of MVPA at 12 months *p* < 0.05, not found to be significant at 6 months) [64]; and a pulmonary rehabilitation high intensity interval training (HIIT) programme (increase to 3200 steps per day versus 740 steps per day at 12 months (*p* = 0.005), not found to be significant at 12 weeks) [63]. 

Two of the RCTs did not report between-group differences, but reported within-group differences (pre- to post-change) for the intervention arms [59,60,65]. These interventions involved: a multi-component educational programme without positive affect and self-affirmation techniques (increase to 415 Kcal per week at 12 months, *p* = 0.02) [59,60]; and an aerobic and resistance training programme with education (increase to 2654 min of total physical activity METs per week (*p* ≤ 0.05), and 1200 min of vigorous physical activity METs per week (*p* ≤ 0.01)) [65]. The non-RCT found that an exercise circuit training programme improved self-reported time spent participating in vigorous physical activity in the asthma group, compared to baseline (data not reported) [67]. Studies that reported no significant intervention effects on physical activity were an aerobic exercise training programme with education [62], and a tailored community-based walking prescription programme [66]. 

Two out of three studies that assessed sedentary behaviour as an outcome reported significant within-group differences. An aerobic and resistance training programme with education reduced sitting time by 120 min at 12 weeks compared to baseline (*p* ≤ 0.05) [65], and a tailored community-based walking prescription programme reduced sedentary time at week 1, 2, 3, 4, 5, and 6 (*p* ≤ 0.05) [56]. The remaining study, a weight loss programme with aerobic and resistance muscle training, did not report a decrease in time spent being sedentary [58,66].

## 4. Discussion

**Figure 3:**
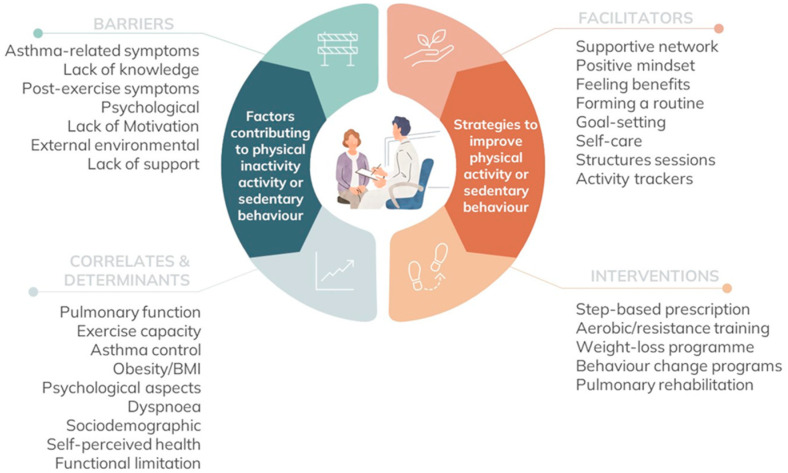
Summary of key considerations when addressing physical inactivity and sedentary behaviour in people with asthma.

This review highlights what is needed to change physical activity from a patient perspective, what factors may contribute to physical inactivity from a research perspective, and what interventions have been shown to be effective for optimising physical activity and sedentary behaviour in people with asthma (Figure 3). There were five main findings: (1) limited research has been done on sedentary behaviour in people with asthma; (2) in addition to common barriers to physical activity experienced by the general population, people living with asthma experience disease-specific barriers including asthma-related symptoms, exacerbations, and comorbidities; (3) having a safe asthma-specific physical activity programme and a supportive network are potential facilitators of physical activity; (4) in line with patient-reported barriers, asthma-specific factors have been shown to be correlates/determinates of physical activity, including pulmonary function, asthma control and dyspnoea in addition to health-related and psychosocial factors; (5) different types of interventions, including structured aerobic and resistance training programmes, have been shown to be effective for increasing physical activity participation, but only a single RCT demonstrated long-term behaviour change.

### 4.1. Physical Activity Barriers and Facilitators

People with asthma mention their condition as the most common barrier to physical activity participation. For instance, fear of exacerbation, or worsening of symptoms during exercise, and feeling embarrassed when exhibiting asthma symptoms have been reported as barriers by people with asthma [33]. People with more severe asthma were also shown to be more likely to believe that exercise can be dangerous [36]. Similar findings have been shown in people with chronic obstructive pulmonary disease (COPD), for whom fear of breathlessness and frequency of exacerbations are common barriers [68]. These fears might occur in people with asthma due to lack of knowledge or to an erroneous understanding of exercising with asthma, another frequently mentioned barrier identified by our review [33,37]. It has previously been shown that parents of children with asthma discourage participation in physical activity due to fears about exercise-induced bronchospasms [69]. Thus, people with asthma may develop early misperceptions that physical activity is not safe for them. 

Another barrier to physical activity mentioned by people with asthma was the experience of unpleasant physical symptoms due to engaging in exercise, including fatigue and pain. To date, no studies have investigated the effects of exercise on fatigue in people with asthma; however, exercise training has shown a positive effect on reducing fatigue in people with COPD [70]. It is likely that people with asthma have impaired physical capacity, which limits their ability to participate in MVPA [71]. People with asthma have reported that exercise is too difficult [34], and have doubts about their capability to physically exert themselves [37]. While supervised exercise training is likely to lead to improved physical capacity [72], a starting point for people with asthma who are exercise-naïve might be to focus on light intensity physical activity within tolerable symptom limits. Physical activity intensity and volume can then be increased over time. The presence of comorbidities should also be considered when addressing post-exercise symptoms, particularly for those with severe asthma. Although it is not reported in the included studies, there is a high prevalence of comorbidities for people with asthma; unpleasant physical symptoms could discourage persons with asthma from engaging in exercise [73].

Psychological issues and lack of motivation were also mentioned as barriers to physical activity [33,34,36]. Applying behaviour change techniques (BCT) as part of a physical activity programme could help people with asthma overcome these barriers and facilitate physical activity. A behaviour change programme has been shown to improve physical activity (time spent in MVPA) in people with asthma, supporting the use of BCT in this population [74]. However, this study assessed physical activity only in the short term; it is unknown if improvements are maintained in the long term [74]. The BCTs implemented as part of a physical activity intervention should be theory-informed, meaning that this decision should be guided by understanding the possible influences of the behaviour [75]. In studies included in this review, people with asthma reported BCTs including goal setting, social support, habit formation and activity trackers as facilitators of physical activity [35,36,38]. 

For people with asthma, support from healthcare professionals in the form of a partnership was highlighted as a facilitator of physical activity [33,36]. This is consistent with identified barriers to physical activity related to healthcare experiences, unclear medical advice, and mistrust of doctors taking care of their asthma [33]. Clinician knowledge about how and what guidance to provide to people with asthma regarding physical activity may also be limited. Data from an international survey suggests that 33% of clinicians had no training on communicating exercise guidance to people with asthma, and 95% agreed that doctors require additional training in preventive care [76]. North American physicians’ main barriers to physical activity counselling were limited time during consults, prioritisation of other health behaviours, and a lack of knowledge on how and where to refer patients for exercise [77]. This highlights the need for healthcare providers to be provided with the resources and education to deliver exercise and physical activity recommendations and to be aware of how to refer patients to specialists, which reinforces the importance of including this information in guidelines. 

### 4.2. Correlates and Determinants of Physical Activity and Sedentary Behaviour

Physical activity was associated with pulmonary (mainly forced expiratory volume in the first second) and extrapulmonary factors in asthma, including exercise capacity and self-reported functional limitation. This corroborates the “can do, do do” concept, which groups people based on their level of physical capacity (can do or can’t do) and their actual physical activity participation (do do or don’t do). In a previous study of people with asthma, 30% were classified as “can’t do, don’t do” while 29% were “can do, do do” [71]. In other words, most people with asthma were physically active if they had retained physical capacity. This highlights the importance of improving pulmonary and physical function for people with asthma to enable and facilitate participation in physical activity. This can be achieved by optimisation of pharmacological therapy and through non-pharmacological approaches like pulmonary rehabilitation. However, few people with asthma are referred to or have access to pulmonary rehabilitation [78], and international guidelines [27] do not include pulmonary rehabilitation as a recommended non-pharmacological approach, despite mounting evidence for the benefits of supervised exercise in this population [55].

In addition to physical capacity, other health-related factors have been shown to be associated with physical activity. In the study by Dogra et al. [41], older males with asthma and ‘excellent self-perceived health’ were 5.39 times more likely to be physically active than older males with asthma who had ‘poor self-perceived health.’ In addition, females who were members of a volunteer organisation were 59% more likely to be physically active than those who were not [41]. Across the studies [22,47,48], physical activity was also associated with age, pulmonary function, anxiety, dyspnoea and BMI. This is similar to what has been shown in people with COPD, in whom physical activity participation is associated with exercise capacity and dyspnoea [79]. These findings support the benefits of models of care in people with asthma, which identify and target traits or phenotypes associated with reduced physical activity participation as a strategy to enable physical activity participation [11,80]. 

FeNo was the only outcome correlated with sedentary behaviour in studies included in our review. However, the strength of these correlations was weak and, as with all other correlations, it is not possible to infer causality. Blood eosinophils was not correlated with sedentary behaviour in two studies; therefore, this relationship remains inconclusive. Although there is an absence of sedentary behaviour research in people with asthma, in the general population, time spent sedentary has shown to be related to being older, female, and physically inactive, smoking, eating high-calorie foods, and engaging in higher mobile phone use [81]. Further, people with COPD who are sedentary have reduced exercise tolerance and participate in less physical activity [82]. These collective findings may assist in addressing sedentary behaviour in people with asthma. 

### 4.3. Interventions for Optimising Physical Activity and Sedentary Behaviour

The systematic review by Tyson et al. [56] included RCTs that increased participation in physical activity in the short term (3 months). These interventions involved a step-based prescription programme using a pedometer, a weight loss programme with aerobic and resistance muscle training and a weight loss lifestyle intervention. The meta-analysis from McLoughlin et al. [54] found an overall significant improvement in steps per day post-intervention in favour of the physical activity intervention (k = 3, n = 142) [57,58,61,62]. This improvement was largely driven by two interventions, the step-based prescription programme using a pedometer, and the weight loss programme with aerobic and resistance muscle training [57,58,61]. However, the step-based prescription programme was not found to be effective in the long term (6–7 months). A HIIT pulmonary rehabilitation intervention included in the reviews by Osadnik et al. [55] and McLoughlin et al. [54] was reported to be effective at improving physical activity at 12 months, but was not found to be effective in the short term [63]. 

The overview of systematic reviews highlights the fact that effective interventions for promoting physical activity are largely heterogeneous, making it difficult to identify effective intervention components. Importantly, only a single intervention (HIIT pulmonary rehabilitation, with supervised exercise delivered 3 times per week for 12 weeks) demonstrated significant improvements in physical activity at long-term follow-up. This may indicate that physical activity interventions to date have failed to consider and address the broad range of factors beyond physical capacity that pose a barrier to physical activity in people with asthma. This review has identified common patient-reported barriers and facilitators to physical activity participation and research-identified correlated and determinants. These are largely about the impact of asthma and non-asthma symptoms and the patient’s ability or perceived ability to safely participate in physical activity while living with these symptoms. No study included within these systematic reviews explicitly reported addressing these factors as part of their physical activity intervention. 

It is important to remember that physical activity is a behaviour, and changing behaviour requires a multidimensional approach to understand how and under what circumstances change is achieved [83,84]. The UK Medical Research Council’s framework for development of behaviour change interventions identifies the importance of involving key stakeholders to co-develop programme theory, choose the most useful research perspective and overcome practical obstacles [84]. Tyson et al. [56] identified action planning, goal setting (behaviour), instruction on how to perform behaviour, demonstration of behaviour, and behavioural practice/rehearsal as the most commonly used BCTs in interventions that were effective. However, these BCTs were also common in interventions that did not effectively increase physical activity. For future studies, researchers should include key stakeholders when designing interventions, and in addition to key BCTs, consider and target the barriers and facilitators reported by people living with asthma [85,86].

In terms of sedentary behaviour, only Tyson et al. [56] reported two interventions that were effective in reducing sedentary time. An aerobic/resistance training programme reduced sitting time by 120 min (compared to baseline) [65]. This is promising; however, siting time was assessed using self-report questionnaires, which have been shown to have poor validity [87,88]. The community-based walking programme analysed differences between device measures of sedentary time, and therefore it cannot be concluded that this intervention is effective for reducing sedentary time [66]. Overall, we cannot determine the types of interventions that effectively optimise sedentary behaviour in people with asthma. How to improve sedentary behaviour in people with asthma, which requires a different approach to targeting physical inactivity, is an important focus for future research.

### 4.4. Summary: Addressing Physical Inactivity

Our review shows that in addition to well-known social and behavioural factors, there are asthma-specific barriers to address in people with asthma to enable physical activity. We recommend a personalised approach in which a prescribed multi-component programme includes components targeted at individualised factors contributing to physical inactivity. According to barriers and facilitators raised by people with asthma, education should be included as part of a multi-component physical activity programme. Education should be clear and focused on asthma and exercise-related topics, and should explain the benefits of physical activity and its safety, self-monitoring skills, how to prevent or manage asthma related symptoms during physical activity, and exercise while living with comorbidities [26]. When physical capacity is identified as a barrier to physical activity for people with asthma, supervised exercise training with peers may be an important step toward enabling physical activity, e.g., pulmonary rehabilitation [72,89]. Strategies chosen should target the lack of motivation that people with asthma experience by setting goals, providing activity trackers, and forming habits [35,36,38]. Outcomes that improved following the effective multi-component interventions included asthma control, pulmonary function, BMI, exercise capacity and anxiety and depression symptoms [54,56], which were also identified in this review as physical activity correlates and determinants. This emphasises the relevance of approaching physical inactivity as part of asthma treatment/management. Most importantly, this approach recognises the support from healthcare professionals that is needed for people with asthma to facilitate physical activity. 

### 4.5. Strengths, Limitations, and Future Research

This narrative review involved a comprehensive search of the available literature to address three aims. It is the first to review (1) barriers and facilitators of physical activity and sedentary behaviour, (2) correlates and determinants of physical inactivity and sedentary behaviour, and (3) effective interventions for optimising physical activity and sedentary behaviour. While a comprehensive and systematic search of the literature was conducted, this was not a systematic review, and therefore methodological bias was not assessed. The risk of bias was not assessed for studies included to address aim 3, and therefore the precision of interventions for optimising physical activity and sedentary behaviour may be limited. A meta-analysis was also not performed. The targeted population of this review was people with asthma; however, how asthma was defined varied across studies. Asthma was defined either objectively (presence of airflow limitation) or according to whether it was diagnosed by a physician, the patient reported ever being told that they had asthma, or the patient was part of an online asthma community. Additionally, the inconsistent reporting of asthma severity across the various studies limits the generalisability of our conclusions. 

## 5. Conclusions

This narrative review provides key considerations when addressing physical inactivity in people with asthma. Consideration should be given to well-known social and behavioural physical activity factors. Addressing the disease-specific barriers reported by people with asthma and providing a safe asthma-specific physical activity programme with a supportive network should be considered. In line with what people with asthma report, asthma-specific factors shown to be correlates/determinates of physical activity included pulmonary function, asthma control and dyspnoea in addition to health-related and psychosocial factors. These findings suggest that multidimensional factors need to be considered when addressing physical inactivity in people with asthma. As such, treatment requires a personalised programme that incorporates strategies targeting asthma-related factors of physical inactivity. There was minimal evidence to provide recommendations for optimising sedentary behaviour in asthma, and more research is needed on the topic. However, due to the known deleterious health effects of sedentary behaviour, it should be addressed in people with asthma. 

## Figures and Tables

**Table 2:** Summary of correlates and determinants of physical activity.

	Pulmonary Function	Exercise Capacity	Asthma Control	Body Mass Index	Eosinophilic Inflammation	Airway Inflammation	Systemic Inflammation	Psychological Aspects	Dyspnoea	Exacerbations	Sociodemographic	Quality of Life	Self-Perceived Health	Self-Reported Functional limitation	Medication Use	Fruit and Vegetable Intake
Mälkiä and Impivaara (1998) [39]	+															
Mancuso et al. (2007) [40]		+														
Dogra et al. (2008) [41]				−				×			+		+	−		
Ramos et al. (2015) [42]		+														
van’t Hul (2016) et al. [19]	×		+													
Good et al. (2017) [43]				−				−		×	+		+			+
Vermeulen et al. (2017) [44]													×			
Yamazaki et al. (2017) [45]	+						×									
Coelho et al. (2018) [46]		×	×					×			×	×				
Hennegrave (2018) [47]	+	+		−				−	−		+	×				
Cordova-Rivera et al. (2019) and Cordova-Rivera et al. (2021) [22,48]	+	+			×		+		−							
Abdo et al. (2021) [49]	+		+	−												
Almatruk and Axon (2022) [50]											+		+	−		
Hansen et al. (2022) [51]	×		×		×		×					×				
Ozsoy et al. (2022) [52]		+														
Ricketts et al. (2023) [53]	+	+	+	−	×	−		−	−			+			−	

× = Was not associated with physical activity; + An increase was associated with physical activity; − A decrease was associated with physical activity.

## Supplementary Materials

The following supporting information can be downloaded at https://www.mdpi.com/article/10.3390/jcm12185998/s1: Figure S1. SANRA reporting checklist; Table S1. Search strategy (Reference [90] is cited in the Supplementary Materials).
