# Supplementary material for: Key Considerations When Addressing Physical Inactivity and Sedentary Behaviour in People with Asthma
Source: J Clin Med. 2023 Sep 15;12(18):5998. doi: 10.3390/jcm12185998 (PMC10531510; doi:10.3390/jcm12185998)
Supplement: Supplementary file 1 [file jcm-12-05998-s001.zip › jcm-2564957-supplementary.pdf]

Supplementary Material

Scale for the Assessment of Narrative Review Articles – SANRA

Please rate the quality of the narrative review article in question, using categories 0–2 on the following scale. For each aspect of quality, please choose the option which best fits your evaluation, using categories 0 and 2 freely to imply general low and high quality. These are not intended to imply the worst or best imaginable quality.

1) Justification of the article's importance for the readership

The importance is not justified. — 0
The importance is alluded to, but not explicitly justified. — 1
The importance is explicitly justified. — 2

Score: 2

2) Statement of concrete aims or formulation of questions

No aims or questions are formulated. — 0
Aims are formulated generally but not concretely or in terms of clear questions. — 1
One or more concrete aims or questions are formulated. — 2

Score: 2

3) Description of the literature search

The search strategy is not presented. — 0
The literature search is described briefly. — 1
The literature search is described in detail, including search terms and inclusion criteria. — 2

Score: 2

4) Referencing

Key statements are not supported by references. — 0
The referencing of key statements is inconsistent. — 1
Key statements are supported by references. — 2

Score: 2

5) Scientific reasoning

(e.g., incorporation of appropriate evidence, such as RCTs in clinical medicine)

The article's point is not based on appropriate arguments. — 0
Appropriate evidence is introduced selectively. — 1
Appropriate evidence is generally present. — 2

Score: 2

6) Appropriate presentation of data

(e.g., absolute vs relative risk; effect sizes without confidence intervals)

Data are presented inadequately. — 0
Data are often not presented in the most appropriate way. — 1
Relevant outcome data are generally presented appropriately. — 2

Score: 2

Sumscore 12

Fig. 1 SANRA - Scale

Content courtesy of Springer Nature, terms of use apply. Rights reserved.

Supplement Figure S1. SANRA reporting checklist.

Supplement Table S1. Search strategy.

1	moderate persistent asthma/ or severe asthma/ or exercise induced asthma/ or occupational asthma/ or asthma.mp. or mild persistent asthma/ or severe persistent asthma/ or asthma/ or allergic asthma/ or mild intermittent asthma/ or asthmatic.mp. or "bronchial asthma".mp.
2	"sedentary behavio?r".mp. or "sedentary lifestyle"/ or sedentarism.mp. or physical activity/ or exercise/ or physical activit*.mp. or physical inactivity/ or physical inactivit*.mp. or sitting.mp. or sitting/ or reclining.mp. or reclining position/ or lying.mp. or seated.mp. or "screen time".mp. or "screen time"/ or "television viewing"/ or circuit training/ or endurance training/ or moderate intensity continuous training/ or continuous training/ or interval training/ or functional training/ or resistance training/ or training/ or muscle training/ or high intensity interval training/ or "daily life physical activit*".mp. or daily life activity/ or "activit* daily living".mp. or running.mp. or running/ or swimming.mp. or swimming/ or swimming time/ or walking.mp. or walking/ or stair climbing.mp. or stair climbing/
3	"barrier*".mp. or "facilitator*".mp. or "promot*".mp. or "overcom*".mp. or "enabl*".mp.
4	"correlat*".mp. or ("determinant" or "determinants").mp.
5	intervention.mp. or intervention study/ or patient education/ or pulmonary rehabilitation.mp. or pulmonary rehabilitation/ or rehabilitation/ or education program/ or program effectiveness/ or program.mp.
6	limit to (human and english language and (adult <18 to 64 years> or aged <65+ years>))
7	limit to (meta analysis or "systematic review")
8	1 and 2 and 6 and 3 or 4
9	1 and 2 and 5 and 6 and 7
